# Spatial immune profiling complements genomic sequencing in biliary tract cancer: hypothesis-generating use cases

**DOI:** 10.1016/j.esmogo.2026.100318

**Published:** 2026-03-23

**Authors:** M. Barsch, I. Godbole, P. Metzger, M. Benzing, L. Klaas, A. Decker, A. Dammer, J. Weiß, E.-P. Dopfer, L. Gräßel, J. Kuehn, C. Miething, H. Becker, S. Lassmann, P. Bronsert, A.M. Schultheis, M. Werner, M. Boerries, J. Duyster, R. Thimme, M. Hofmann, M. Quante, B. Bengsch

**Affiliations:** 1 Clinic for Internal Medicine II, Gastroenterology, Hepatology, Endocrinology and Infectious Disease, Medical Center—University of Freiburg, Faculty of Medicine, University of Freiburg, Freiburg, Germany; 2 Institute of Medical Bioinformatics and Systems Medicine, Medical Center—University of Freiburg, Faculty of Medicine, University of Freiburg, Freiburg, Germany; 3 Department of Diagnostic and Interventional Radiology, Medical Center—University of Freiburg, Faculty of Medicine, University of Freiburg, Freiburg, Germany; 4 Comprehensive Cancer Center Freiburg, Medical Center—University of Freiburg, Faculty of Medicine, University of Freiburg, Freiburg, Germany; 5 Institute for Surgical Pathology, Medical Center—University of Freiburg, Faculty of Medicine, University of Freiburg, Freiburg, Germany; 6 Clinic for Hematology and Medical Oncology, University Medical Center Goettingen, Goettingen, Germany; 7 Department of Medicine I, Medical Center—University of Freiburg, Faculty of Medicine, University of Freiburg, Freiburg, Germany; 8 German Cancer Consortium (DKTK) Partner site Freiburg, a partnership between DKFZ and Medical Center—University of Freiburg, Heidelberg, Germany; 9 German Cancer Research Center (DKFZ), Heidelberg, Germany; 10 Signalling Research Centres BIOSS and CIBSS, University of Freiburg, Freiburg, Germany

**Keywords:** biliary tract cancer, precision oncology, molecular tumor board, imaging mass cytometry, tumor immune microenvironment, BAP1

## Abstract

**Background:**

Personalized therapy approaches targeting homologous recombination deficiency (HRD) in biliary tract cancer (BTC) remain poorly explored with limited clinical data available. Beyond BRCA1/2, the therapeutic implications of BRCA1-associated protein 1 (BAP1) alterations are unclear.

**Materials and methods:**

We describe three patients with BTC and HRD-related mutations as hypothesis-generating use cases. We illustrate how integration of multiplexed imaging mass cytometry-based spatial immune profiling with genomic sequencing may help generate testable biological hypotheses beyond genomics alone.

**Results:**

A patient with germline *BRCA2*-mutated BTC together with a positive HRD score was sensitive to platinum-based therapy and poly (ADP-ribose) polymerase inhibition. In contrast, a patient with loss of *BAP1* was linked to a CD8+ T-cell enriched tumor microenvironment, classified as spatially immune enriched and responded long-term to subsequent immune checkpoint inhibition and tyrosine kinase inhibition therapy.

**Conclusion:**

These observations provide a rationale for integrated genomic and spatial immune profiling in BTC, which will require further prospective validation.

## Highlights

• IMC enables spatial immune profiling complementary to genomics.
• Immune context varied across BTC cases with HRD-associated alterations.
• A *BAP1*-mutated BTC case showed marked intratumoral CD8+ T-cell enrichment.
• Spatial immune profiling complemented MTB treatment discussion.

## Introduction

Biliary tract cancer (BTC), with a 5-year survival rate of <20%, ranks among the leading causes of cancer-related death worldwide. Its poor prognosis is largely associated with late-stage diagnosis and aggressive tumor growth. However, up to 40% of intrahepatic cholangiocarcinoma (iCCA) cases harbor actionable targets, highlighting the importance of molecular testing.^1^^,^^2^ Isocitrate dehydrogenase 1 (*IDH1*) mutations, fibroblast growth factor receptor 2 (FGFR2) fusions, and human epidermal growth factor receptor 2 (HER2) amplifications represent established actionable targets in BTC and their therapeutic relevance has been validated in phase II/III trials.^3^, ^4^, ^5^ In contrast, comparable evidence for less frequent homologous recombination (HR) deficiency (HRD)-associated alterations as a therapeutic biomarker in BTC is lacking.^6^ Throughout this manuscript, we use the term ‘HRD-associated alterations’ to describe genetic alterations in HR or related DNA damage response genes, without implying a uniform HRD phenotype or sensitivity to poly (ADP-ribose) polymerase (PARP) inhibition.

While *BRCA1/2* alterations are well-established therapeutic markers in several tumor types,^7^, ^8^, ^9^ their relevance in BTC is less clearly defined, and the biological and therapeutic implications of *BAP1* loss remain even more ambiguous. *BAP1* is a tumor suppressor gene that is involved in the repair of DNA damage by regulating chromatin structure and interacting with other proteins that are part of HR machinery, such as *BRCA1*.^10^ Loss-of-function mutations represent the most frequent *BAP1* variants^11^; however, considerable heterogeneity exists, as certain destabilizing variants or complex interactions (e.g. via ASXL1) can lead to altered or context-dependent activity.^12^ Therefore, *BAP1* mutations remain challenging to characterize and represent a major obstacle to effective evaluation and targeted therapeutic intervention.

It is possible that loss of *BAP1* and impairment of DNA repair may drive HR defects comparable to *BRCA1/2* and could thus provide a therapeutic rationale for PARP inhibition.^13^ This hypothesis is supported by preclinical models^14^; however, the data on clinical application are highly controversial.^15^, ^16^, ^17^ Other recent studies, however, indicate that *BAP1* alterations are linked to an inflamed tumor immune microenvironment (TIME), which could make these tumors responsive to immunotherapeutic approaches.^18^^,^^19^ Overall, it remains unclear whether patients with loss-of-function *BAP1*-mutated tumor tissues are appropriate candidates for maintenance therapy with PARP inhibitors or immunotherapy. Further investigation and complementary therapeutic strategies are urgently needed.

In this context, molecular tumor boards (MTBs) play a key role in the development of personalized therapy approaches, as they focus on the identification of actionable targets.^20^ Recent findings highlight the influence of the TIME on tumor biology and thus on therapeutic response.^21^ Notably, immune checkpoint inhibitor (ICI)-based combinations have recently demonstrated overall survival benefit in advanced BTC in randomized phase III trials, underscoring the clinical relevance of immune context and the need for improved biomarkers beyond genomics alone.^22^^,^^23^ However, established predictive biomarkers in BTC remain limited. The TIME is frequently categorized into immune-rich (‘hot’) or immune-depleted (‘cold’) tumors, depending on immune cell infiltration and immune checkpoint expression.^24^ To dissect the immunologic landscape of tumor tissue, the use of multiplexed imaging mass cytometry (IMC) provides an innovative method in tumor samples, enabling high-dimensional single-cell analysis with spatial resolution.^21^^,^^25^ In this context, spatial immune evaluation has recently been proposed for the other primary liver cancer, hepatocellular carcinoma (HCC), mapping immune architectures and immunotypes predictive of the efficacy of immunotherapy using deep spatial single-cell profiling of the TIME.^21^ Three major immunotypes were observed, defined by CD8+ T-cell numbers and distribution: immune depleted, compartmentalized, and immune enriched. Importantly, patients with CD8+ T-cell-enriched tumors had significantly longer progression-free survival (PFS) and overall survival.^21^

Despite its clinical relevance, the consideration of TIME in treatment decisions is still lacking in most MTBs, as treatment recommendations are primarily based on genetic findings. Spatial tumor profiling using the existing tissue specimen may close a gap between tumor genetics and immunophenotyping for personalized therapy. Here, we report three BTC patients with genetic alterations associated with HRD as hypothesis-generating use cases in whom immune profiling was carried out in addition to genetic sequencing to illustrate how IMC may serve as a complementary, hypothesis-generating tool alongside genomic profiling in precision gastrointestinal oncology.

## Materials and methods

### Patients

This descriptive case series included BTC patients with HRD-associated alterations who received MTB-guided therapy and had available tumor tissue suitable for IMC analysis. Tumor tissue samples from three patients with histologically confirmed BTC were included via the MTB registry of the Comprehensive Cancer Center Freiburg (CCCF). All patients presented with advanced-stage disease and underwent comprehensive molecular profiling. MTB-guided therapy recommendations were derived from integrated genomic and clinical data. Treatment response was assessed by contrast-enhanced computed tomography or magnetic resonance imaging at ∼12-week intervals and by serial tumor marker measurements. Clinical and molecular characteristics are summarized in Table 1.

**Table 1:** Clinical and molecular characteristics of the study patients

	Patient 1	Patient 2	Patient 3
Sex	Female	Female	Female
Age at first diagnosis (years)	60	69	38
BTC subtype	iCCA	pCCA	iCCA
Extent of disease at first diagnosis	Resectable	Metastasized	Resectable
Histological grading	G2	G2	G2
Extent of disease at MTB presentation	Metastasized	Metastasized	Metastasized
Genetic test	TSO500	TSO500	TSO500 and WES
Tumor material	Liver resection of primary tumor	Resection of peritoneal metastasis	Liver resection of primary tumor (TSO500)/resection of pulmonary metastasis (WES)
Tumor cell count	60%	40%	TSO500: 50% WES: 70%
Type of HR mutation	*BRCA2* (p.A938Pfs∗21)	*BRCA2* (p.L2132∗)	*BAP1* (p.W202∗)
Germline versus somatic	Germline	Somatic	Germline
Allele frequency	70.7%	11.1%	TSO500: 81.9% WES: 90%
Co-mutations	*ARID1A* (p.M1564)	*TP53* (p.L319Rfs∗26)	*NRAS* (p.G12S)
TMB (mut/Mb)	17.3 (high)	4 (low)	TSO500: 7.9 (low) WES: 2.56 (low)
HRD	90	n/a	36 (WES)
Microsatellite status	MSS (1.6% instable loci)	MSS (3.6% instable loci)	MSS (TSO500: 1.64% instable loci; WES: 0.35% instable loci)

Clinical and genomic data of three female patients with biliary tract cancer (BTC), including age at diagnosis, tumor subtype, disease extent, histological grade, and molecular testing results. Genomic profiling was carried out using TruSight Oncology 500 (TSO500) and whole-exome sequencing (WES), assessing homologous recombination (HR)-related gene mutations, tumor mutational burden (TMB), and microsatellite status.

iCCA, intrahepatic cholangiocarcinoma; MTB, molecular tumor board; pCCA, perihilar cholangiocarcinoma.

This study was conducted in accordance with the Declaration of Helsinki and approved by the local Ethics Committee of the University Medical Center Freiburg (reference numbers: 24-1065-S1-retro and 369/19). Written informed consent for molecular analyses and translational research was obtained from all patients at the time of inclusion in the institutional MTB registry.

### Genetic sequencing

Molecular profiling was carried out using next-generation sequencing with the TruSight Oncology 500 (TSO500) panel (Illumina, San Diego, CA) and whole-exome sequencing (WES) on tumor tissue, with matched germline controls when available. DNA was extracted from formalin-fixed, paraffin-embedded tumor samples and peripheral blood. Sequencing achieved a minimum target coverage of 500× for panel sequencing and 100× for WES. Variants were identified using standardized bioinformatic pipelines (see Supplementary Methods, available at https://doi.org/10.1016/j.esmogo.2026.100318).

### Imaging mass cytometry

IMC was carried out on formalin-fixed, paraffin-embedded BTC tissue sections using a validated 42-marker antibody panel covering major adaptive and innate immune cell populations (Supplementary Table S1, available at https://doi.org/10.1016/j.esmogo.2026.100318). Metal-conjugated antibodies were obtained pre-labeled or conjugated in-house using the Maxpar X8 antibody labeling kit (Fluidigm, South San Francisco, CA) according to the manufacturer’s instructions. Antibodies were titrated and validated on control liver and tonsil tissue.

Tissue preparation, antigen retrieval, staining, and image acquisition were carried out essentially as previously described,^25^^,^^26^ with full methodological details provided in the Supplementary Methods, available at https://doi.org/10.1016/j.esmogo.2026.100318. Regions of interest (ROIs) were selected by pathologists based on corresponding hematoxylin–eosin immunohistochemical staining, covering tumor core, invasive margin, and adjacent non-tumorous tissue. IMC data were acquired on a Hyperion Imaging System (Fluidigm) at a spatial resolution of 1 μm^2^ per pixel. Raw data were preprocessed using CyTOF software (v7.0) (Standard BioTools, South San Francisco, CA) and visualized using MCD Viewer (Fluidigm) and FIJI/ImageJ (NIH, Bethesda, MD).

### Spillover compensation and image processing

To correct for low-level signal interference between neighboring mass channels, global spillover compensation was applied to the acquired data using the CATALYST package in R, following the approach described by Chevrier et al.^27^ Spillover-corrected data were processed using an adapted steinbock pipeline^28^ including hot-pixel filtering and single-cell segmentation with Mesmer based on nuclear, membrane, and cytoplasmic markers, preserving spatial cell coordinates.

### Image normalization

Image channels were normalized using PENGUIN (PErcentile Normalisation GUI Image deNoising).^28^ This method scales signal intensities between 0 and 1, removes background noise, and applies a percentile-based approach to reduce marker-specific signal noise. Percentiles and thresholds were manually determined for each channel using sample images and then applied consistently across all ROIs.

### Single-cell analysis

Further analysis was carried out in R (version 4.2.2) (R Foundation for Statistical Computing, Vienna, Austria). Basic code was used from the Bodenmiller Github repository to extract spatial experiment information from preprocessed data. PhenoGraph clustering was carried out using the Rphenograph package (k = 30) based on normalized marker expression channels listed in Supplementary Table S1, available at https://doi.org/10.1016/j.esmogo.2026.100318. Statistical testing was carried out at the single-cell level where appropriate (two-sided *t*-test or Wilcoxon rank-sum test, depending on distribution). No formal statistical comparisons were carried out between patients due to the limited sample size (*n* = 3). Accordingly, all patient-level results are presented descriptively.

### Cell density

Cell densities per cell type were calculated by normalizing cluster-based single-cell counts to the analyzed tissue area per ROI (cells/mm^2^). If more than one ROI per patient existed, the mean was calculated. Single-cell counts from defined cell types (e.g. CD8+ T cells) were exported as an Excel table for downstream visualization and bar-plot representation in GraphPad Prism (version 10) (GraphPad Software, San Diego, CA).

### Spatial immunotype classification

Spatial immunotype classification was employed as previously described.^21^ Briefly, the calculated CD8+ T-cell density for the entire tumor tissue was first assessed. Samples with fewer than 200 CD8+ T cells/mm^2^ were classified as immune depleted. For samples exceeding this threshold, the distribution of CD8+ T cells between tumor parenchyma and tumor stroma was evaluated. Cytomapper package in R was used to extract location-based information. Parenchymal and stromal CD8+ T-cell densities as well as their ratios were calculated for each ROI as described in the ‘Cell density’ section. When the ratio of parenchymal to stromal CD8+ T-cell density exceeded 0.6, the tissue was classified as immune enriched; ratios <0.6 were classified as immune compartmentalized. The spatial immune classification framework was applied in an exploratory manner based on the data from a different primary liver cancer entity. This approach has not been validated for BTC and was used for comparative, hypothesis-generating purposes.

### Clinical and molecular characterization of BTC patients with HRD-associated alterations

**Figure 1:**
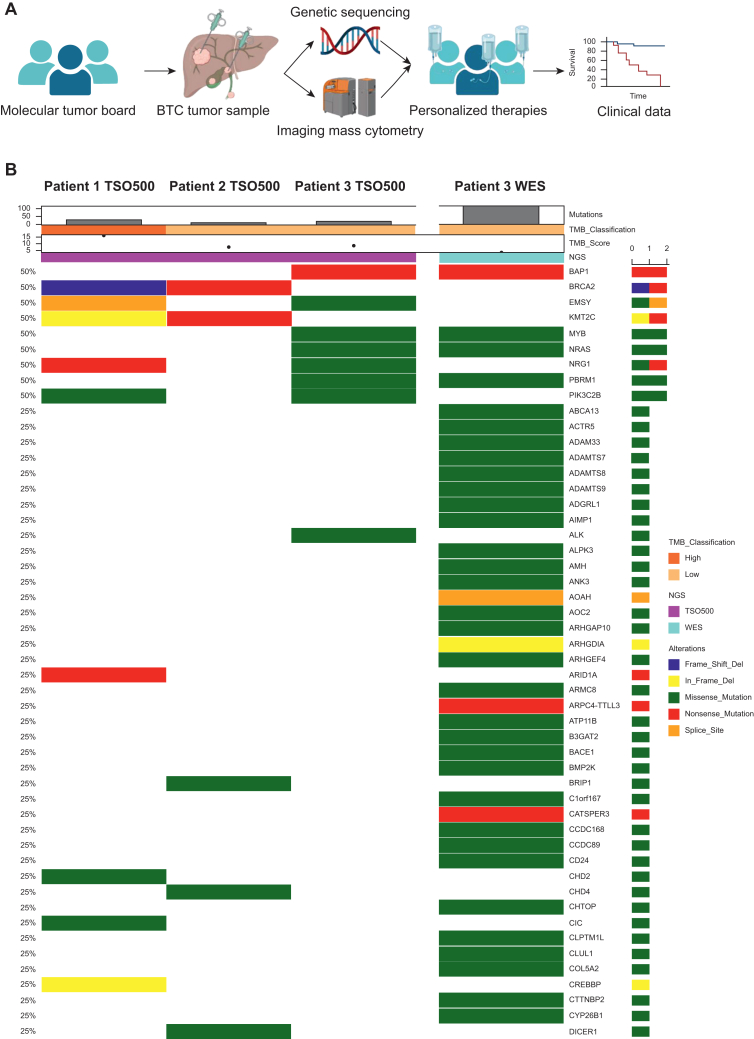
**Study workflow and genomic landscape of three biliary tract cancer (BTC) patients included in the molecular tumor board (MTB).** (A) Study workflow. Three patients with BTC were enrolled in the MTB of the Comprehensive Cancer Center Freiburg (CCCF). Tumor tissue was collected and used for genomic sequencing and multiplexed imaging mass cytometry (IMC) on corresponding tissue sections. Based on the integrated molecular and spatial immune data, personalized therapy recommendations were made by the MTB and clinical outcomes were documented. (B) Genomic landscape of the three BTC patients. Oncoplot depicting somatic alterations detected by targeted next-generation sequencing (tNGS, TruSight Oncology 500, TSO500) and, for patient 3, additional whole-exome sequencing (WES). Columns 1-3 display TSO500 data of patients 1-3, while column 4 shows the WES results of patient 3. Differences in detected variants and allele frequencies reflect both methodological coverage and potential temporal tumor evolution. The upper annotation tracks show sequencing modality, tumor mutational burden (TMB, mutations per megabase), and TMB classification (low versus high). Each column represents an individual patient, and each row corresponds to a gene. Color coding indicates the type of genetic alteration, including frameshift deletions (blue), in-frame deletions (yellow), missense mutations (green), nonsense mutations (red), and splice-site variants (orange). Bars on the right summarize mutation frequencies across the analyzed genes. The plot highlights *BRCA2* and *BAP1* alterations as key homologous recombination deficiency-related variants and displays co-occurring mutations such as *ARID1A*, *TP53*, and *NRAS*.

Comprehensive molecular profiling using the TSO500 panel and/or WES was carried out in three patients with metastatic BTC (Figure 1A and B; Table 1).

Patient 1 was a 62-year-old female with iCCA who developed metastatic disease after resection and adjuvant capecitabine. Molecular analysis of the primary tumor identified a pathogenic germline *BRCA2* truncating variant (p.A938Pfs∗21), associated with a high HRD score (genomic instability score 90), elevated tumor mutational burden (TMB; 17.3 mut/Mb), and microsatellite stability (MSS) (Figure 1B; Tables 1 and 2).

**Table 2:** Genetic variants detected in the study cohort

Gene	OG/TSG	Chr	Coordinate	HGVSc	HGVSp	AF (%)	ClinVar	VICC	Clinical trials
BRCA2∗ (https://www.oncokb.org/gene/BRCA2	TSG	chr13	32911297	NM_000059.3: c.2806_2809del (https://www.oncokb.org/gene/BRCA2/A938Pfs∗21)	NP_000050.2: p.Ala938Profs∗21 (https://www.genomenexus.org/variant/13:g.32911300_32911303del)	70.7	P	https://search.cancervariants.org/#BRCA2	https://clinicaltrials.gov/search?cond=BRCA2
BRCA2∗ (https://www.oncokb.org/gene/BRCA2)	TSG	chr13	32914887	NM_000059.3:c.6395T>G (https://www.oncokb.org/gene/BRCA2/(p.L2132∗))	NP_000050.2:p.Leu2132∗ (https://www.genomenexus.org/variant/13:g.32914887T%3EA)	11.1	P	https://search.cancervariants.org/#BRCA2	https://clinicaltrials.gov/search?cond=BRCA2
BAP1 (https://www.oncokb.org/gene/BAP1)	TSG	chr3	52440899	NM_004656.4:c.605G>A (https://www.oncokb.org/gene/BAP1/W202∗)	NP_004647.1:p.Trp202∗ (https://www.genomenexus.org/variant/3:g.52440899C%3eT)	81.9		https://search.cancervariants.org/#BAP1	https://clinicaltrials.gov/ct2/results?cond=BAP1+W202X&term=&cntry=&state=&city=&dist=

Summary of identified genetic variants affecting oncogenes (OGs) and tumor suppressor genes (TSGs). The table lists the affected gene, chromosomal location, nucleotide and protein alterations [according to Human Genome Variation Society. (HGVS) nomenclature], allele frequency (AF), and corresponding links to clinical databases and trial information. Variants were classified for pathogenicity using ClinVar and evaluated according to Variant Interpretation for Cancer Consortium (VICC) guidelines.

Patient 2, a 69-year-old female with metastatic perihilar cholangiocarcinoma, underwent molecular profiling of peritoneal and ovarian metastases. A somatic truncating *BRCA2* variant (p.L2132∗) and a co-occurring *TP53* frameshift mutation were identified. The tumor exhibited low TMB (4 mut/Mb) and MSS status (Figure 1B; Tables 1 and 2).

Patient 3, a 37-year-old female with recurrent metastatic iCCA, was found to harbor a germline truncating *BAP1* mutation (p.W202∗). This alteration was confirmed by both TSO500 and WES carried out on tumor tissue from different metastatic sites, indicating a stable BAP1-driven molecular profile (Figure 1B; Tables 1 and 2).

In summary, molecular profiling revealed distinct HRD-associated alterations across the three BTC cases, providing the basis for subsequent MTB-guided personalized treatment strategies.

### IMC reveals a strong CD8+ T-cell-enriched tumor microenvironment in the BAP1-mutated BTC patient

**Figure 2:**
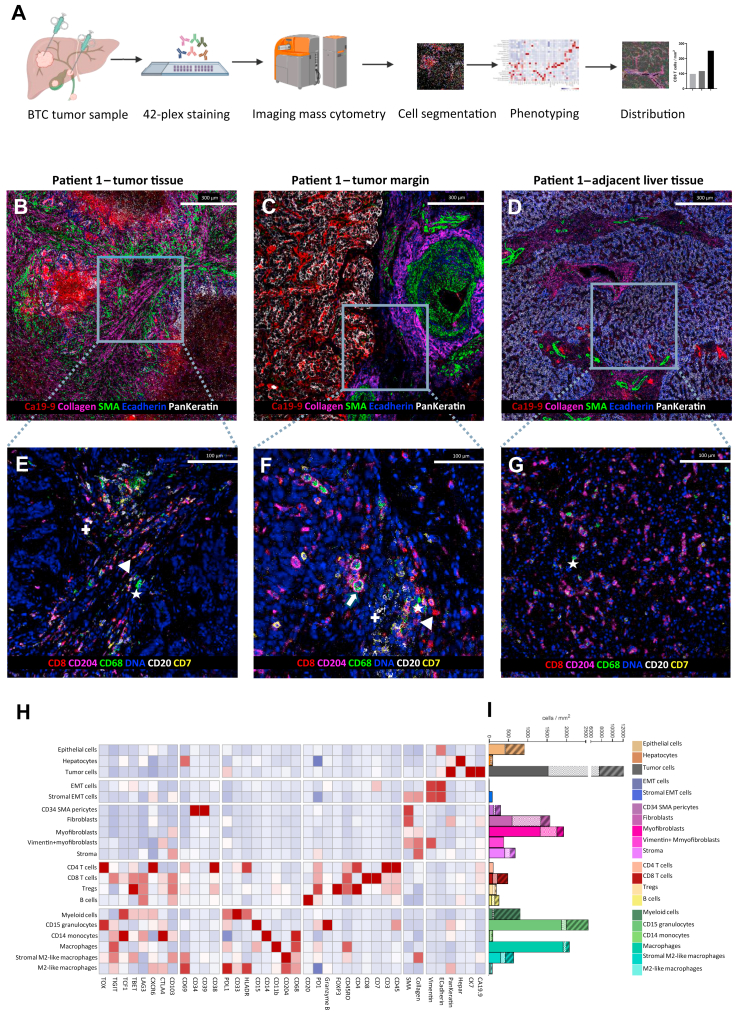
**Spatial immune architecture of biliary tract cancer (BTC) revealed by imaging mass cytometry (IMC).** (A) Workflow of the IMC analysis. Tumor samples were processed through a 42-marker IMC staining panel, followed by high-resolution image acquisition, single-cell segmentation, cell phenotyping, and spatial distribution analysis. (B-D) Representative IMC images showing different tissue compartments from BTC tumor tissue: tumor (B), tumor margin (C), and adjacent healthy liver tissue (D). CA19-9 identifies tumor cells; collagen and α-smooth muscle actin (α-SMA) mark stromal components; E-cadherin and pankeratin delineate hepatic parenchyma. Scale bars: 300 μm. (E-G) Enlarged IMC images highlighting immune cell populations within the corresponding compartments: (E) tumor, (F) tumor margin, (G) adjacent healthy liver tissue. CD8+ T cells, CD68+ macrophages, CD68+ CD204+ M2-like macrophages, CD7+ natural killer (NK) cells, and CD20+ B cells are predominantly detected within the tumor and its invasive front, but are scarce in the adjacent liver parenchyma. Scale bars: 100 μm. (H) PhenoGraph clustering of all IMC-derived single-cell data from the three BTC patients (tumor tissue). The heatmap displays 20 distinct cell phenotypes across tumor, stromal, and immune compartments. (I) Adjacent bar plots indicate cell densities (cells/mm^2^): solid-colored bars represent patient 1, dotted bars indicate patient 2, and striped bars correspond to patient 3. The color legend summarizes marker-defined cell populations included in the clustering. EMT, epithelial–mesenchymal transition.

To dissect the tumor immune landscape in these patients, we utilized a highly multiplexed spatial profiling approach using IMC (Figure 2A).

Such an approach was recently used to accurately inform about the ICI treatment-associated spatial immune architecture of the other primary liver cancer entity, HCC.^21^ We hypothesized that it might also inform about the immune component of the TIME in BTC and therefore comprehensively profiled the immune architecture across different tumor immune compartments—tumor tissue (Figure 2B), tumor margin (Figure 2C), and healthy adjacent liver (Figure 2D)—using corresponding sections of tumor tissue that were also used for genetic sequencing. Representative IMC images are shown (Figure 2B-G). Following 42-plex IMC staining (Supplementary Table S1, available at https://doi.org/10.1016/j.esmogo.2026.100318), CA19-9-positive tumor cells were identified in the tumor core and at the tumor margin (Figure 2B and C). The tumor stroma was characterized by collagen- and α-smooth muscle actin-positive myofibroblasts, whereas adjacent liver tissue showed preserved hepatic parenchyma (Figure 2D). CD8+ T cells were present within the tumor and at the margin but were scarce in adjacent liver tissue (Figure 2E-G). Macrophages (CD68+) were detected in tumor and margin regions, with M2-like subsets (CD68+CD204+) predominantly localized at the tumor margin. B cells (CD20+) were present in tumor and margin areas but largely absent from non-tumorous liver tissue (Figure 2E-G).

**Figure 3:**
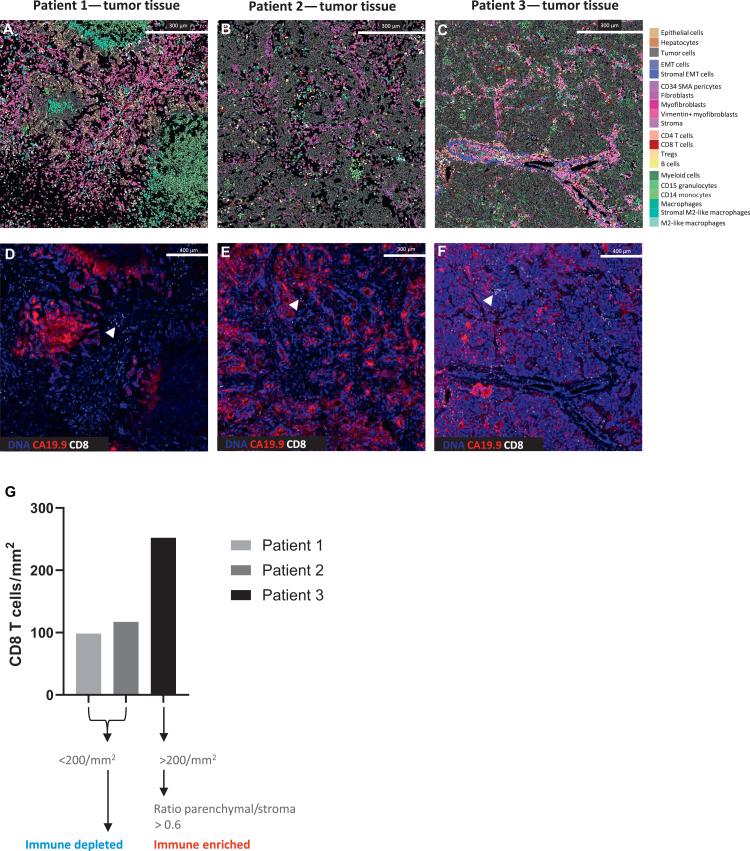
**Spatial CD8+ T-cell enrichment distinguishes *BAP1*- from *BRCA*-mutated biliary tract cancer.** (A-C) Representative imaging mass cytometry (IMC) overlays of tumor tissue from patient 1 (A), patient 2 (B), and patient 3 (C). Colors indicate the distinct cell populations identified by PhenoGraph clustering, as defined in Figure 2H. Scale bars: 300 μm. (D-F) Corresponding IMC images of the same tumor regions highlighting CD8+ T cells (white) and CA19-9-positive tumor cells (red). Arrowheads indicate representative examples of CD8+ T cells for orientation, while additional CD8+ T cells are distributed throughout the depicted areas. A markedly higher intratumoral CD8+ T-cell density adjacent to CA19-9-positive tumor cells is observed in *BAP1*-mutated patient 3 (F) compared with *BRCA2*-mutated patient 1 (D) and patient 2 (E). Scale bars: 400 μm (D, F); 300 μm (E). (G) Bar plots show mean cell densities (cells/mm^2^) across four tumor regions of interest (ROIs) per patient; images depict representative ROIs. Patient 3 shows a markedly higher intratumoral CD8+ T-cell density in the tumor core (mean across four tumor ROIs: 252.08 cells/mm^2^), whereas patient 1 and patient 2 exhibit lower densities (mean across four tumor ROIs: 98.46 and 117.09 cells/mm^2^, respectively). Based on the ratio of parenchymal to stromal CD8+ T-cell distribution (>0.6), the sample of patient 3 (*BAP1*-mutated) was classified as immune enriched, while patients 1 and 2 (*BRCA2*-mutated) were classified as immune depleted, according to the spatial immune framework described by Salié et al.^21^ SMA, smooth muscle actin.

We analyzed the BTC tumor tissue by comparing the immune populations of the two *BRCA2*-mutated patients with the *BAP1*-mutated patient. We utilized a semi-supervised machine learning-aided analysis pipeline to identify key adaptive and innate immune cell populations in a data-driven manner. PhenoGraph clustering of single-cell data from tumor regions identified 20 distinct cellular phenotypes, including adaptive immune subsets (CD4+ and CD8+ T cells, B cells) and innate immune populations (macrophages, M2-like macrophages, natural killer cells, granulocytes, and antigen-presenting cells) (Figure 2H). Notably, we observed a higher density of CD8+ T cells (mean across four tumor ROIs) within the tumor tissue of *BAP1*-mutated patient 3 (252.08 cells/mm^2^) compared with *BRCA2*-mutated patient 1 (98.46 cells/mm^2^) and patient 2 (117.09 cells/mm^2^) (Figure 2I). We visualized the spatial distribution of identified cell populations within the BTC tumor microenvironment using IMC images of representative tumor regions from each patient (Figure 3A-C).

Figure 3A-C shows corresponding tumor areas from patient 1 (Figure 3A), patient 2 (Figure 3B), and patient 3 (Figure 3C), with cell populations displayed according to the color legend derived from the PhenoGraph clustering shown in Figure 2H. The same tissue regions are depicted in Figure 3D-F, highlighting CD8+ T cells (indicated by arrowheads) in patient 1 (Figure 3D), patient 2 (Figure 3E), and patient 3 (Figure 3F). Arrowheads mark representative examples of CD8+ T cells for illustration, whereas additional CD8+ T cells are present throughout the respective regions. Notably, a markedly higher density of CD8+ T cells adjacent to CA19-9-positive tumor cells was observed in *BAP1*-mutated patient 3 (Figure 3F) compared with *BRCA*-mutated patients 1 and 2 (Figure 3D and E). We compared the CD8+ T-cell infiltration data with recently published findings of a proposed spatial immune classification.^21^ In this work, based on the ratio of parenchymal to stromal CD8+ T-cell densities, liver tumor samples with a CD8+ T-cell density <200 per mm^2^ were classified as immune depleted, while those above this threshold were categorized as immune compartmentalized or immune enriched depending on the distribution of CD8+ T cells between tumor parenchyma and stroma.^21^ Based on the integration of the results from the cohort analyzed in the cited publication, the *BAP1*-mutated tumor tissue was determined as an immune-enriched immunotype, in contrast to the immune-depleted *BRCA2*-mutated patients (Figure 3G). This classification validated for the other major primary liver cancer entity was used as a comparative reference framework and potential surrogate for the immunotherapy response in BTC.

In summary, the comprehensive spatial immune profiling of tumor tissues using IMC revealed differences in the composition of the intratumoral immune landscape, indicating a comparatively higher intratumoral CD8+ T-cell density in the *BAP1*-mutated BTC patient.

### Integrated molecular and spatial immune profiling as hypothesis-generating use cases

**Figure 4:**
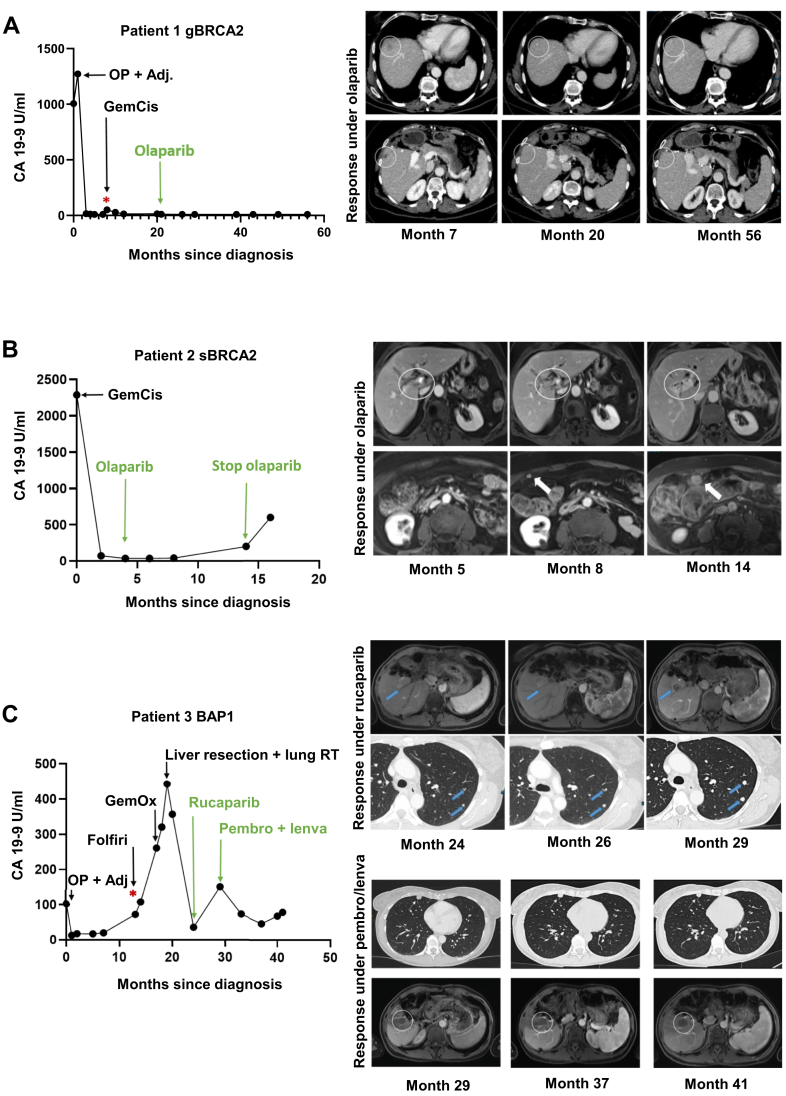
**Molecular tumor board (MTB)-guided therapy responses in three biliary tract cancer (BTC) patients.** (A) Patient 1 with germline BRCA2 (gBRCA2)-mutated BTC. The left graph shows tumor marker dynamics over time since initial diagnosis, including initial surgery (OP), followed by recurrence (asterisk) and subsequent therapy lines: platinum-based chemotherapy gemcitabine/cisplatin (GemCis) and poly(ADP-ribose) polymerase (PARP) inhibition (olaparib) as recommended by the MTB. Right-sided, computed tomography (CT) images depict regression of intrahepatic lesions during olaparib treatment (months 20-44) after prior response to GemCis (months 7-20). (B) Patient 2 with somatic BRCA2 (sBRCA2) mutation. The left graph illustrates tumor marker progression and therapy timeline, including gemcitabine/cisplatin and subsequent olaparib treatment. CT images on the right demonstrate radiological progression under olaparib with a new central liver lesion and progressive peritoneal carcinomatosis (months 5-15). (C) Patient 3 with *BRCA1*-associated protein 1 (BAP1) mutation. The left graph shows tumor marker trends and applied therapies, including primary resection and adjuvant chemotherapy (OP + Adj.) (gemcitabine/cisplatin), FOLFIRI, gemcitabine/oxaliplatin, surgical resection of liver metastases, radiotherapy (RT) of pulmonary lesions, rucaparib, and subsequently pembrolizumab plus lenvatinib (pembro + lenva) as recommended by the MTB. The asterisk denotes tumor recurrence. CT scans (top right) depict disease progression during rucaparib treatment (months 24-29), whereas follow-up images (bottom right) demonstrate stable liver and lung metastases under pembrolizumab + lenvatinib (months 29-41).

The genetic and immunological analyses conducted through the MTB at CCCF resulted in personalized treatment recommendations after prior standard therapies. Based on the identified *BRCA* and *BRCA*-related alterations indicating HRD, and in the absence of other targeted treatment options at evaluation, the MTB recommended a PARP inhibitor for all patients, including the one with a *BAP1* mutation. PARP inhibitors exploit HR defects and have shown clinical activity in HRD-positive tumors.^7^^,^^9^^,^^29^ In the case of patient 1 with germline *BRCA2* variant (p.A938Pfs∗21) and a positive HRD score of 90, this treatment was initiated. As shown in Figure 4A, the patient first underwent primary tumor resection plus adjuvant chemotherapy (‘OP + Adj.’), followed by a subsequent tumor recurrence (indicated by an asterisk in Figure 4A). After this recurrence, the patient received platinum-based chemotherapy (gemcitabine/cisplatin), to which she responded favorably, and subsequently achieved a durable long-term response to the PARP inhibitor olaparib. Remarkably, this response has been sustained for >36 months to date, indicating a long-term benefit and supporting that PARP inhibition is highly effective in controlling platinum-sensitive BTC with pathogenic germline *BRCA* mutation. Clinical benefit from platinum-based chemotherapy and PARP inhibition in germline *BRCA2*-mutated BTC aligns with the established efficacy in germline *BRCA*-associated cancers^7^, ^8^, ^9^ (ovarian, pancreatic, prostate) and is included for tumor-agnostic context. In contrast, patient 2 carrying a somatic *BRCA2* variant (p.L2132∗) showed progression after 12 months on olaparib (Figure 4B). Furthermore, no clinical benefit was observed in patient 3 with *BAP1* mutation (p.W202∗). Before rucaparib initiation, the patient had undergone primary tumor resection with adjuvant therapy (‘OP + Adj.’) and later showed progression under folinic acid, fluorouracil, and irinotecan and gemcitabine/oxaliplatin. The transient decline in tumor markers immediately before rucaparib was attributable to surgical resection of liver metastases and radiotherapy (RT) of pulmonary lesions (‘liver resection + lung RT’) (Figure 4C). After initiation of rucaparib therapy, the patient experienced rapid progression of both liver and lung metastases, leading to treatment discontinuation after 12 weeks (Figure 4C). We identified a CD8+ T-cell-enriched tumor microenvironment through the additional spatial immune profiling by IMC (Figure 3G); thus, an immunotherapy-based regimen was considered in the context of the MTB, supported by the observed immune-enriched phenotype. ICIs are used in first-line therapy for advanced BTC, supporting immunotherapy-based approaches in this entity.^22^^,^^23^ Building on this concept, pembrolizumab plus lenvatinib has been explored beyond the first-line setting, providing a clinical rationale for its use in selected refractory cases.^30^^,^^31^ This combination achieved disease control for the first time after failure of all prior standard treatments, with stable disease for 12 months to date (Figure 4C).

## Discussion

This study represents a descriptive case series with detailed biomarker analysis. Within this exploratory framework, we first contextualize our observations using *BRCA1/2*-altered BTC, for which enhanced sensitivity to platinum-based chemotherapy and PARP inhibitors has been reported, supported by tumor-agnostic studies and phase III trials in other cancers.^7^, ^8^, ^9^ These and our findings suggest germline *BRCA2* mutations together with a positive HRD score as potential predictive biomarkers, warranting molecular testing during first-line therapy to guide PARP inhibitor maintenance in BTC. Nevertheless, definitive clinical validation in BTC is still pending. In contrast to well-characterized *BRCA1/2* alterations—such as the pathogenic truncating *BRCA2* variants detected in our cohort (c.2806_2809del; p.Ala938Profs21 and c.6395T>G; p.Leu2132, Table 2)—*BAP1* mutations represent a biologically heterogeneous group and do not uniformly confer HRD.

To identify an appropriate personalized therapy approach for the *BAP1*-mutated BTC patient, we conducted a comprehensive analysis of the tumor microenvironment as a hypothesis-generating use case, focusing on the patient-specific immune landscape alongside genomic sequencing. In contrast to the *BRCA*-mutated tissue samples, the *BAP1*-mutated tumor tissue exhibited a strong enrichment of CD8+ T cells (Figure 3G). In BTC and other tumor entities, the presence of CD8+ T cells within the tumor microenvironment is generally considered as a favorable indicator for better outcome^32^ and response to ICI.^33^ However, systematic data quantifying and comparing CD8+ T-cell densities in BTC tumor tissue using a validated score of immune infiltrates to predict response to ICI are still lacking. In addition, validated predictive biomarkers for ICI response in BTC remain limited. In particular, programmed death-ligand 1 expression and TMB have shown variable and overall insufficient predictive performance across studies.^34^^,^^35^ In our *BAP1*-mutated case, disease control under pembrolizumab plus lenvatinib was observed despite low TMB, underscoring the limitations of these biomarkers in selected BTC patients.

Recently, a spatial immune evaluation was proposed for another subtype of primary liver tumor—HCC—mapping the spatial immune types predictive of immunotherapy by carrying out deep spatial single-cell profiling and bioinformatic deconvolution of the immune architecture of HCC tumor microenvironment using IMC.^21^ Related approaches are known from colorectal cancer.^36^ Three main patterns of immune cell infiltration were observed in HCC, defined by different quantity and distribution of infiltrating CD8+ T cells between the tumor parenchyma and the stroma: an immune-depleted, a compartmentalized, and an immune-enriched immunotype. PFS during ICI therapy varied considerably across the spatial immune types, with improved survival of CD8+ T-cell-enriched patients.^21^ Applying a similar approach, our IMC-based spatial analysis categorized the *BAP1*-mutated BTC as immune enriched, consistent with the strong CD8+ T-cell infiltration observed and with the patient’s favorable clinical response to ICI in combination with tyrosine kinase inhibition therapy (off-label via the MTB) (Figure 4C). The observed association between CD8+ T-cell enrichment and response to pembrolizumab plus lenvatinib in the *BAP1*-mutated case is observational and *post hoc* and does not establish causality. Nevertheless, this observation supports further investigation of spatial immune classifications as a complementary approach to genomic profiling in BTC.

### Conclusion

This hypothesis-generating case series illustrates how integration of genomic and spatial immune profiling may generate testable hypotheses in BTC, particularly in biologically ambiguous contexts such as *BAP1* alterations. While platinum sensitivity and PARP inhibitor benefit in germline *BRCA2*-mutated BTC are consistent with existing evidence, the spatial immune characterization of the *BAP1*-mutated case—showing marked intratumoral immune cell enrichment, including increased CD8+ T-cell density—was observed alongside disease control under subsequent immunotherapy-based regimen with pembrolizumab plus lenvatinib. These findings are exploratory and require validation in larger, dedicated cohorts.

## Declaration of generative AI and AI-assisted technologies in the writing process

We used an AI-assisted writing tool (ChatGPT, OpenAI) for English language polishing and organization. All scientific content, data interpretation, and conclusions were generated and verified by the authors.
