# Supplementary material for: Spatial immune profiling complements genomic sequencing in biliary tract cancer: hypothesis-generating use cases
Source: ESMO Gastrointest Oncol. 2026 Mar 23;12:100318. doi: 10.1016/j.esmogo.2026.100318 (PMC13049665; doi:10.1016/j.esmogo.2026.100318)
Supplement: Supplementary Data [file mmc1.docx]

**Spatial immune profiling complements genomic sequencing in biliary tract cancer: hypothesis-generating use cases**

Maryam Barsch^1,✉^, Ira Godbole^1^, Patrick Metzger^2^, Maximilian Benzing^1^, Lioba Klaas^1^, Annegrit Decker^1^, Anna Dammer^1^, Jakob Weiß^3^, Elaine-Pashupati Dopfer^4,5^, Linda Gräßel^4,6,7^, Julia Kuehn^4,7^, Cornelius Miething^4,7^, Heiko Becker^4,7,8^, Silke Lassmann^4,5,8^, Peter Bronsert^5^, Anne Maria Schultheis^4,5^, Martin Werner^4,5,8^, Melanie Boerries^2,4,8,9^, Justus Duyster^4,7,8^, Robert Thimme^1^, Maike Hofmann^1^, Michael Quante^1*^, Bertram Bengsch^1,8,10*^

Affiliations

1Clinic for Internal Medicine II, Gastroenterology, Hepatology, Endocrinology and Infectious Disease, Medical Center—University of Freiburg, Faculty of Medicine, University of Freiburg, Freiburg, Germany

2Institute of Medical Bioinformatics and Systems Medicine, Medical Center—University of Freiburg, Faculty of Medicine, University of Freiburg, Freiburg, Germany

3Department of Diagnostic and Interventional Radiology, Medical Center - University of Freiburg, Faculty of Medicine, University of Freiburg, Freiburg, Germany

4Comprehensive Cancer Center Freiburg, Medical Center—University of Freiburg, Faculty of Medicine, University of Freiburg, Freiburg, Germany

5Institute for Surgical Pathology, Medical Center—University of Freiburg, Faculty of Medicine, University of Freiburg, Freiburg, Germany

6Clinic for Hematology and Medical Oncology, University Medical Center Goettingen, Goettingen, Germany

7Department of Medicine I, Medical Center—University of Freiburg, Faculty of Medicine, University of Freiburg, Freiburg, Germany

8German Cancer Consortium (DKTK) Partner site Freiburg, a partnership between DKFZ and Medical Center—University of Freiburg, Heidelberg, Germany

9German Cancer Research Center (DKFZ), Heidelberg, Germany

10Signalling Research Centres BIOSS and CIBSS, University of Freiburg, Freiburg, Germany.

*Equally contributing

^✉^Corresponding author:

Maryam Barsch, MD

University Hospital Freiburg

Hugstetter street 55

79106 Freiburg

[maryam.barsch@uniklinik-freiburg.de](mailto:maryam.barsch@uniklinik-freiburg.de)

**Supplementary Methods**

**Genetic sequencing**

Molecular genetic characterization of the tumors was performed using NGS with the TSO500 panel and WES from Illumina. Sequencing was conducted on Illumina NextSeq and NovaSeq platforms. Prior to sequencing, DNA was primarily extracted from formalin-fixed, paraffin-embedded (FFPE) tumor tissue samples and from EDTA blood for matching control samples. The quality and quantity of the extracted DNA were assessed using Qubit and Bioanalyzer analyses to ensure that the sequencing samples met the required purity criteria. After library preparation and hybrid capture enrichment, sequencing was performed with a target coverage of at least 500x for panel sequencing and 100x for WES. The bioinformatic processing of sequencing data followed a standardized analysis pipeline. First, raw data underwent quality control using FastQC , followed by adapter trimming and removal of low-quality reads with Trimmomatic^1^. The sequences were then aligned to the human reference genome (GRCh37) using BWA-MEM^2^, with further processing performed using SAMtools^3,4^ and picard tools. Variant calling was conducted with GATK Mutect2^5^. For annotation of the identified variants, Ensembl VEP^6,7^ and SnpEff^8^ were used to determine the biological consequences of individual mutations. Statistical and post-processing analyses were performed in R 4.4.2^9^ and visualizations were generated using the maftools R package (version 2.2.0)^10^.

**Imaging mass cytometry**

IMC antibody panel: A 42-marker IMC panel with markers for major adaptive and innate immune cell types was designed and validated (Supplementary Table 1). Metal-labeled antibodies were either obtained pre-conjugated (Fluidigm) or labeled in house by conjugating purified antibodies to lanthanide metals using the Maxpar X8 antibody labelling kit (Fluidigm) according to the manufacturer’s instructions. In addition, 89-Yttrium (III) nitrate tetrahydrate (Sigma Aldrich) and 157-Gadolinium (III) chloride (Trace Sciences Int.) were obtained, diluted in L-buffer to a 1M stock solution and further diluted to a 50 µM working solution for subsequent antibody labelling with the Maxpar X8 labelling kit. 20µL Cell-ID™ Cisplatin-194Pt (Fluidigm) was used for antibody labeling of histone H3. Metal-conjugated antibodies were titrated and validated on liver and tonsil tissue. Sample preparation and staining for IMC: Samples were stained essentially as previously described^11,12^. Briefly, BTC tissue sections were incubated at 60°C for one hour, dewaxed in Xylene twice for 15 minutes and rehydrated in descending concentrations of ethanol (100% - 100% - 95% - 80%) for five minutes each, then rinsed in TBS (pH 7.6) for 10 minutes. Epitope retrieval was performed in a decloaking chamber (Biocare Medical) with EnVision FLEX Target Retrieval Solution High pH for 30 minutes at 95°C. Slides were cooled down to RT for 20 minutes inside the buffer and 10 minutes in TBS. Tissue sections were encircled with a PAP pen and blocked for 45 minutes at room temperature using SuperBlock (TBS) Blocking Buffer. The sections were then stained with a mix of metal-labeled primary antibodies diluted in TBS with 0.5% BSA and incubated at 4°C overnight. Slides were rinsed in TBS-T (TBS supplemented with 0.2% Tween-20) twice and twice in TBS for 5 minutes each. Tissue sections were then stained with Iridium Cell-ID intercalator (500 µM, 1:2000) in TBS for 30 minutes at room temperature. Slides were rinsed three times for 5 minutes in TBS, dipped in ddH2O for 5 seconds and air-dried. Slides were stored at RT until image acquisition. Image acquisition: Briefly, tuning of the instrument was performed according to the manufacturer’s instructions. Regions of interest were determined using H&E stains. Tissue sections were laser-ablated spot-by-spot at 200 Hz resulting in a pixel 3 size/resolution of 1 µm². Preprocessing of the raw data was conducted using the CyTOF software v7.0 (Fluidigm). Image acquisition control was performed using MCD Viewer v1.0.560.6 (Fluidigm). A 2.25 mm² image was acquired using a Hyperion Imaging System (Fluidigm). Image visualizations were made with FIJI, a distribution of ImageJ (v1.52p). Images were acquired as mcd and .txt files.

**Supplementary Table 1. IMC antibody panel.**

List of metal-conjugated antibodies used for imaging mass cytometry (IMC), including metal isotope tag, target antigen, antibody clone, company/source, catalogue ID, applied antibody volume per 1000 µL master mix, and final dilution.

| **Metal** | **Target** | **Clone** | **Company** | **ID** | **Volume (µl / 1000 µl)** | **Dilution** |
| --- | --- | --- | --- | --- | --- | --- |
| 113In | CD68 | KP1 | Biolegend | 916104 | 2.5 | 1:400 |
| 115In | HLA-DR | TAL-1B5 | Abcam | ab176408 | 2.5 | 1:400 |
| 141Pr | SMA | 1A4 | Novus Biologicals | NBP2-34522-0.1MG | 10 | 1:100 |
| 142Nd | CD15 | HI98 | Biolegend | 301902 | 2.5 | 1:400 |
| 143Nd | CTLA4 | SP355 | Abcam | ab238271 | 20 | 1:50 |
| 144Nd | CD69 | EPR21814 | Abcam | 234512 | 5 | 1:200 |
| 145Nd | CD3 | D7A6E | CellSignalling | 85061BF | 10 | 1:100 |
| 146Nd | Ki-67 | B56 | BD Bioscience | 556003 | 2.5 | 1:400 |
| 147Sm | CD14 | EPR3653 | Abcam | ab226121 | 10 | 1:100 |
| 148Nd | Pan-Keratin | C11 | CellSignalling | 17171SF | 1.25 | 1:800 |
| 149Sm | Hepar | GR3391991-2 | CellSignalling | 96422S | 5 | 1:200 |
| 150Nd | PD-L1 | 22C3 | Dako (Agilent) | M3653 | 10 | 1:100 |
| 151Eu | CXCR6 | Polyclonal | Invitrogen | PA5-33462 | 5 | 1:200 |
| 152Sm | TCF-1 | C6309 | BioLegend | 655202 | 2.5 | 1:400 |
| 153Eu | TOX | NAN448E | CellSignalling | 81229SDF | 1.25 | 1:800 |
| 154Sm | Ca19.9 | 121SLE | Abcam | ab3982 | 2.5 | 1:400 |
| 155Gd | FoxP3 | 236A/E7 | invitrogen | 14-4777-82 | 2.5 | 1:400 |
| 156Gd | CD4 | EPR6855 |  | ab181724 | 1.25 | 1:800 |
| 157Gd | Lag3 | EPR4392 | Abcam | 369302 | 0.5 | 1:2000 |
| 158Gd | TIGIT | BLR047F | BioLegend | ab243903 | 1.25 | 1:800 |
| 159Tb | CD11b | EPR1344 | abcam | ab52632 | 5 | 1:200 |
| 160Gd | T-bet | 4B10 | abcam | 644802 | 5 | 1:200 |
| 161Dy | CD20 | H1 | Biolegend | 555677 | 2.5 | 1:400 |
| 162Dy | CD8a | C8/144B | BD Bioscience | 372902 | 0.625 | 1:1600 |
| 163Dy | TROP2 | EPR20043 | Biolegend | ab271996 | 6.6 | 1:150 |
| 164Dy | Claudin 18.2 | 43-14A | Abcam | ab314691 | 5 | 1:200 |
| 165Ho | PD-1 | D4W2J | Abcam | 63815 | 5 | 1:200 |
| 166Er | CD204 | J5HTR3 | CST | 14-9054-82 | 5 | 1:200 |
| 167Er | GranzymeB | EPR20129-217 | Invitrogen | 372202 | 2.5 | 1:400 |
| 168Er | CD39 | EPR20627 | Biolegend | ab236038 | 5 | 1:200 |
| 169Tm | Collagen | Polyclonal | Abcam | 3169023D | 1.25 | 1:800 |
| 170Er | CD103 | SP301 | Fluidigm | ab245746 | 10 | 1:100 |
| 171Yb | CD7 | EPR4242 | Abcam | ab230834 | 0.4 | 1:2500 |
| 172Yb | CD38 | EPR4106 | Abcam | AB176886 | 1.25 | 1:800 |
| 173Yb | CD45RO | UCHL1 | Abcam | 3173016D | 0.625 | 1:1600 |
| 174Yb | CD33 | SP266 | Fluidigm | ab238784 | 5 | 1:200 |
| 175Lu | CD34 | EP373Y | Abcam | ab198395 | 1.25 | 1:800 |
| 176Yb | CK7 | RCK105 | Abcam | BM6003P | 1.25 | 1:800 |
| 194Pt | HH3 | D1H2 | Origene | 60932SF | 2.5 | 1:400 |
| 195Pt | Vimentin | D2K6W | CellSignalling | 46173SF | 10 | 1:100 |
| 196Pt | E-Cadherin | 24 E10 | CellSignaling | 96743SF | 1.25 | 1:800 |
| 89Y | CD45 | D9M8I | CellSignalling | 13917T | 2.5 | 1:400 |

**References**

1. Bolger, A. M., Lohse, M. & Usadel, B. Trimmomatic: a flexible trimmer for Illumina sequence data. *Bioinformatics* **30**, 2114–2120 (2014).

2. Li, H. & Durbin, R. Fast and accurate short read alignment with Burrows-Wheeler transform. *Bioinformatics* **25**, 1754–1760 (2009).

3. Danecek, P. *et al.* Twelve years of SAMtools and BCFtools. *Gigascience* **10**, giab008 (2021).

4. Li, H. *et al.* The Sequence Alignment/Map format and SAMtools. *Bioinformatics* **25**, 2078–2079 (2009).

5. Van der Auwera, G. A. *et al.* From FastQ data to high confidence variant calls: the Genome Analysis Toolkit best practices pipeline. *Curr Protoc Bioinformatics* **43**, 11.10.1-11.10.33 (2013).

6. Dyer, S. C. *et al.* Ensembl 2025. *Nucleic Acids Res* **53**, D948–D957 (2025).

7. McLaren, W. *et al.* The Ensembl Variant Effect Predictor. *Genome Biol* **17**, 122 (2016).

8. Cingolani, P. *et al.* A program for annotating and predicting the effects of single nucleotide polymorphisms, SnpEff: SNPs in the genome of Drosophila melanogaster strain w1118; iso-2; iso-3. *Fly (Austin)* **6**, 80–92 (2012).

9. R Core Team. R: A Language and Environment for Statistical Computing. R Foundation for Statistical Computing (2022).

10. Mayakonda, A., Lin, D.-C., Assenov, Y., Plass, C. & Koeffler, H. P. Maftools: efficient and comprehensive analysis of somatic variants in cancer. *Genome Res* **28**, 1747–1756 (2018).

11. Barsch, M. *et al.* T-cell exhaustion and residency dynamics inform clinical outcomes in hepatocellular carcinoma. *J Hepatol* **77**, 397–409 (2022).

12. Schwabenland, M. *et al.* Deep spatial profiling of human COVID-19 brains reveals neuroinflammation with distinct microanatomical microglia-T-cell interactions. *Immunity* **54**, 1594-1610.e11 (2021).
